# miR-146a-5p, miR-223-3p and miR-142-3p as Potential Predictors of Major Adverse Cardiac Events in Young Patients with Acute ST Elevation Myocardial Infarction—Added Value over Left Ventricular Myocardial Work Indices

**DOI:** 10.3390/diagnostics12081946

**Published:** 2022-08-12

**Authors:** Alina Ioana Scărlătescu, Teodora Barbălată, Anca Volumnia Sima, Camelia Stancu, Loredan Ștefan Niculescu, Miruna Mihaela Micheu

**Affiliations:** 1 Department of Cardiology, “Carol Davila” University of Medicine and Pharmacy, 050474 Bucharest, Romania; 2 Department of Cardiology, Clinical Emergency Hospital of Bucharest, 014461 Bucharest, Romania; 3 Lipidomics Department, Institute of Cellular Biology and Pathology “Nicolae Simionescu” of the Romanian Academy, 8, B.P. Hasdeu Street, 050568 Bucharest, Romania

**Keywords:** STEMI, young, MACE, myocardial work indices, miRNA

## Abstract

Acute ST elevation myocardial infarction (STEMI) remains a leading cause of morbidity and mortality worldwide despite continuous advances in diagnostic, prognostic and therapeutic methods. Myocardial work (MW) indices and miRNAs have both emerged as potential prognostic markers in acute coronary syndromes in recent years. In this study we aim to assess the prognostic role of myocardial work indices and of a group of miRNAs in young patients with STEMI. We enrolled 50 young patients (<55 years) with STEMI who underwent primary PCI and 10 healthy age-matched controls. We performed standard 2D and 3D echocardiography; we also calculated left ventricular global longitudinal strain (GLS) and the derived myocardial work indices. Using RT-PCR we determined the plasmatic levels of six miRNAs: miR-223-3p, miR-142-3p, miR-146a-5p, miR-125a-5p, miR-486-5p and miR-155-5p. We assessed the occurrence of major adverse cardiac events (MACE) at up to one year after STEMI. Out of 50 patients, 18% experienced MACE at the one-year follow-up. In a Cox univariate logistic regression analysis, myocardial work indices were all significantly associated with MACE. The ROC analysis showed that GWI, GCW and GWE as a group have a better predictive value for MACE than each separately (AUC 0.951, *p* = 0.000). Patients with higher miRNAs values at baseline (miR-223-3p, miR-142-3p and miR-146a-5p) appear to have a higher probability of developing adverse events at 12 months of follow-up. ROC curves outlined for each variable confirmed their good predictive value (AUC = 0.832, *p* = 0.002 for miR-223-3p; AUC = 0.732, *p* = 0.031 for miR-142-3p and AUC = 0.848, *p* = 0.001 for miR-146a-5p); the group of three miRNAs also proved to have a better predictive value for MACE together than separately (AUC = 0.862). Moreover, adding each of the miRNAs (miR-233, miR-142-3p and miR-146a-5p) or all together over the myocardial work indices in the regression models improved their prognostic value. In conclusion, both myocardial work indices (GWI, GCW and GWE) and three miRNAs (miR-223-3p, miR-142-3p and miR-146a-5p) have the potential to be used as prognostic markers for adverse events after acute myocardial infarction. The combination of miRNAs and MW indices (measured at baseline) rather than each separately has very good predictive value for MACE in young STEMI patients (C-statistic 0.977).

## 1. Introduction

Ischaemic heart disease remains to this day a leading cause of morbidity and mortality worldwide [1,2]. Although myocardial infarction is considered a disease occurring in older adults, in recent years its prevalence in the young population has increased [3,4]. So far, there has been no standard definition of “young” age in patients with STEMI. Previous studies used different age thresholds, varying from <30 years to <55 years [5,6,7]. We decided to use the broader cut off and included in our study adult patients, younger than 55 years old.

Patients with an acute coronary syndrome at a young age are at high risk for other future major cardiovascular events; therefore, an optimal follow-up and preventive strategies are of paramount importance in this population category [8] Considering the significant health and socioeconomic burden of AMI in younger patients, the discovery of new markers is essential to improve the diagnosis, risk stratification and prediction of adverse events in this group.

### 1.1. Myocardial Work Indices

Left ventricular systolic function is one of the most important and used prognostic marker after STEMI. It is traditionally expressed by left ventricular ejection fraction (LVEF), based on systolic and diastolic volume changes. However, LVEF is subjective, there are technical limitations and inconsistency, impaired reproducibility and a high interobserver variability [9]. Myocardial strain analysis has been developed as a more comprehensive technique for the evaluation of LV function enabling the assessment of global and regional myocardial deformation during the cardiac cycle [10]. Moreover, prior studies have advocated that LV GLS measured after STEMI has incremental prognostic value over LVEF [11,12]. Unfortunately, like LVEF, LV GLS is afterload-dependent, especially in patients with impaired left ventricular function, but to a lesser extent [13].

Echocardiographic assessment of myocardial work may further improve the evaluation of myocardial function. This non-invasive method is based on a standardised LV pressure curve adjusted to arterial pressure. Myocardial work parameters offer an estimation of LV systolic function taking into account loading conditions; therefore, this method is very promising for evaluating the failing heart and predicting prognosis [14,15]. There are four indices that can be assessed noninvasively with 2D speckle tracking imaging for myocardial work evaluation: global work index (GWI), global contraction work (GCW), global wasted work (GWW) and global work efficiency (GWE). Their prognostic potential has so far been assessed in various cardiovascular pathologies such as mitral regurgitation [16], aortic stenosis [17], advanced heart failure [18] and acute coronary syndromes [19]. In acute myocardial infarction, two studies have shown promising results: Lustosa et al., who tested the long-term prognostic value of GWE in STEMI patients, found that lower GWE values in the acute phase were associated with worse long-term survival [20] and Butcher et al. concluded that lower GWI values were independently associated with increased all-cause mortality at 6 months of follow-up [21]. However, the research regarding myocardial work indices in STEMI focused mainly on one parameter rather than testing the predictive power of all four indices; moreover, so far, no study has tested these echocardiographic markers in young patients. Therefore, we aim to evaluate the prognostic potential of all myocardial work indices as MACE predictors in young STEMI patients.

### 1.2. microRNAs

In recent years, microRNAs (miRNAs) have appeared as promising diagnostic and prognostic markers involved in the pathophysiology of cardiovascular diseases [22]. Many studies suggest that miRNAs play crucial roles in a variety of essential biological processes, including proliferation, development, differentiation and apoptosis [23]. Their small size, simple chemical composition, high stability, capability to withstand extreme conditions and a cost-effective quantification by RT-PCR make them excellent diagnostic and prognostic markers [22,24]. In addition, many miRNAs are remarkably stable and easily detectable in the peripheral blood [25,26]. The levels of circulating miRNAs are different in specific ways under specific pathological conditions [27,28,29]. This indicates that circulating miRNAs may be excellent candidate diagnostic and prognostic biomarkers of various diseases [30,31].

In this study, we tested six miRNAs (miR-233, miR-142-3p, miR-155-5p, miR-486-5p, miR-125a-5p and miR-146a-5p), known to be associated with coronary artery disease from previous research. MiR-233-3p is almost exclusively of platelet or megakaryocyte origin [32]; the biological activity of miR-223-3p is related to aggregation and granule secretion [32]. It has proved to be a marker of atherosclerotic plaque instability in patients with CAD [33] and also a predictor of thrombotic events that could be used for ischemic risk stratification after AMI [34]. MiR-142-3p plays a role in various inflammatory diseases, such as atherosclerosis [35]. Higher plasmatic levels of miR-142-3p were potential markers to predict MACE in CAD patients after PCI in a study form 2019 [36]. MiR-146a-5p exhibits a protective effect against cardiac ischaemia/hypoxia-induced apoptosis [37] and is also related to coronary artery disease (CAD) [38,39,40]. miR-146a-5p is also expressed in vascular endothelial cells, smooth muscle cells and monocytes/macrophages, and regulates the development of atherosclerosis by acting on different target genes [41,42,43]. It has proved to be a negative feedback regulator of inflammatory response; it is involved in regulating innate immune responses and its expression in myocardial tissue has been reported to increase with the onset of MI [44]. MiR-125a-5p is thought to regulate macrophage activation, lipid metabolism and the regulation of atherogenesis [45] critical processes in coronary artery disease [46] MiR-486-5p is a muscle-enriched miRNA, found to be upregulated in patients with acute coronary syndrome; Zhang et al. proved its diagnostic potential in AMI [47]. MiR-155-5p is an inflammatory-related miRNA, upregulated in activated inflammatory cells; it modulates immune responses via cell differentiation and function and inflammatory cytokine secretion [48,49]. In mice, the decreased expression of the miR-155-5p has been associated with enhanced atherosclerosis, decreased plaque stability and decreased T cell regulation [50].

Considering all the above, we hypothesised that both myocardial work parameters and the six miRNAs might play a role in predicting outcome patients with acute coronary syndromes; therefore, we propose to assess their value as prognostic markers in young patients (<55 years) with STEMI.

## 2. Materials and Methods

### 2.1. Study Population

**Figure 1:**
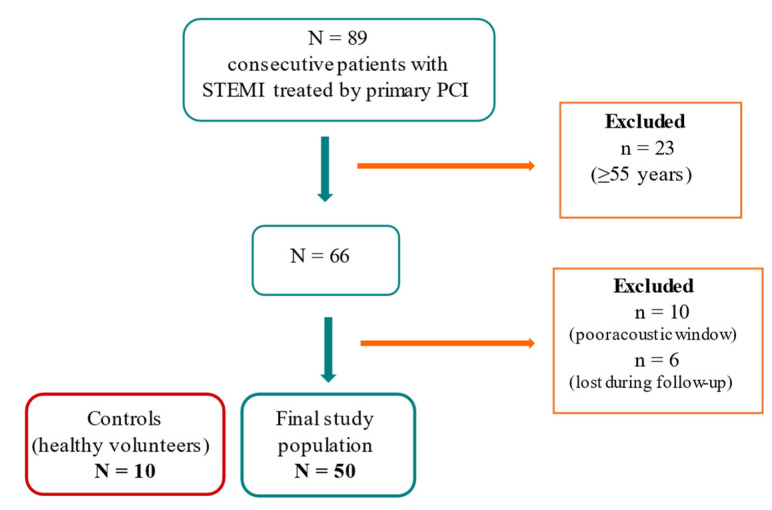
Inclusion flow chart of the study.

We enrolled in this prospective study young patients (<55 years old) with STEMI admitted to our hospital (in 2019–2020) and treated by primary PCI. We first selected 89 consecutive STEMI patients, excluded 23 of them for being >55 years, 10 were excluded due to poor acoustic window and 6 were lost during follow-up, leaving a final study group of 50 (Figure 1). We also chose 10 age-matched controls (healthy volunteers) for the miRNA results validation.

Patients with previous myocardial infarction or cardiac surgery, recent stroke (within six months), recent surgery or trauma (within 6 months), active malignancy or autoimmune diseases, chronic renal failure (eGFR < 30 mL/min/1.73 m^2^), chronic liver failure (defined as a Child–Pugh score of 3), chronic respiratory failure (defined as PO_2_ < 50 mmHg and/or PCO_2_ > 50 mmHg), patients with addictions, poor compliance or those who refused to sign the informed consent were excluded.

This study was approved by the Ethics Committee from the Clinical Emergency Hospital of Bucharest and all patients signed an informed consent form at enrolment.

### 2.2. Echocardiography

We performed 2D standard echocardiography in all patients included in this study using a GE VIVID E9 ultrasound system, both at baseline and at follow-up. Baseline evaluation was made within 5 days after admission. At six months of follow-up, we assessed the evolution in time of the echocardiographic parameters.

The recordings and measurements were performed in accordance with the European [51] and American [52] echocardiographic guidelines. An offline data analysis was done by two independent operators experienced in echocardiography using EchoPAC software.

Besides the conventional parameters, we also measured the LV global longitudinal strain and LV mechanical dispersion using the speckle tracking technique and also 3D left ventricle echocardiography, for a better quantification of the ventricular function.

#### Myocardial Work Analysis

Myocardial work indices were calculated from LV pressure–strain loops by integrating the LV strain data and noninvasively estimating the LV pressure (considered to be equal to arterial blood pressure measured with a brachial cuff sphygmomanometer) [14,20].

The quantification of noninvasive myocardial work was performed using a commercially available software package (EchoPAC, GE Medical Systems).

LV strain data were acquired using 2D speckle-tracking echocardiography by manually tracing the LV endocardial border in the apical long-axis, 2 and 4-chamber views. The peak LV pressure was measured using the patient’s brachial cuff blood pressure recordings with the peak systolic LV pressure assumed to be equal to the peak arterial pressure. An LV pressure–strain curve was then automatically constructed using a reference curve provided by the software package and adjusted to the different cardiac cycle phases using valvular event timing (opening and closing timings of the aortic and mitral valves). LV myocardial work was calculated by integrating the product of the rate of segmental shortening and instantaneous LV pressure over time, thus obtaining myocardial work as a function of time during systole and isovolumic relaxation [21].

The following parameters were obtained from this analysis [14,53,54]:

- Global work index (GWI)—the area within the global LV pressure–strain loop (calculated from mitral valve closure to mitral valve opening), representing the total LV work performed in a single cardiac cycle.
- Global constructive work (GCW)—the myocardial work performed during the shortening of a myocardial segment in systole and during lengthening in isovolumic relaxation, representing the total work contributing to the pump function.
- Global wasted work (GWW)—the negative myocardial work performed during the lengthening of a myocardial segment in systole or during shortening in isovolumic relaxation, and which therefore does not contribute to LV ejection.
- Global work efficiency (GWE)—the sum of the constructive work in all LV segments, divided by the sum of the constructive and wasted work in all LV segments; it is expressed as a percentage: GCW/(GCW + GWW).

### 2.3. Blood Sample Collection and Storage

Whole blood samples harvested in EDTA tubes were obtained by peripheral venous puncture in the first 24–48 h after admission for STEMI. All blood samples were collected after primary PCI, in all patients. Plasma was separated from blood samples after centrifuging (1000× *g*) for 15 min at −4 °C within 30 min of collection and then aliquoted in Eppendorf tubes (300 µL each) and frozen at −80 °C immediately.

### 2.4. miRNA Isolation and Quantification

miRNAs were isolated from plasma using the miRNeasy Serum/Plasma kit (Qiagen, Hilden, Germany), following the manufacturer’s instructions. A quantity of 25 fmol of synthetic cel-miR-39 was added to each sample during miRNA purification as previously described [38].

The plasmatic levels of (hsa)-miR-223-3p (ID 002295), hsa-miR-146a-5p (ID 000468), hsa-miR-486-5p (ID 001278), hsa-miR-125a-5p (ID 002198), hsa-miR-142-3p (ID 000464) and hsa-miR-155-5p (ID 002623) were determined by TaqMan technology. Reverse-transcription was performed with a pool of TaqMan miRNA-specific stem-loop primers on a Veriti PCR system. Real-time quantitative PCR was performed using the hydrolysis probes of miRNA TaqMan assays on a ViiA7 real-time PCR system (Applied Biosystems, Thermo Fisher Scientific, Waltham, MA, USA) and for each sample, triplicate measurements were done on 384-well reaction plates.

The data were analysed using ViiA7 Software v1.2 with the automatic Cq setting. The expression level of each miRNA was determined relative to that of exogenously added cel-miR-39-2p (ID 000200) as previously reported [55].

### 2.5. Coronary Angiography

According to the European society of cardiology practice guidelines [56], invasive coronary angiography followed by primary PCI was performed at admission in all patients with STEMI from our study. None of the included patients had significant coronary lesions at hospital discharge.

### 2.6. Follow-Up and Outcomes

Patients were followed up for up to one year after STEMI. At 6 months, we performed a more detailed follow-up—clinical examination, standard echocardiography—and at one year, a telephone follow-up.

All included patients were followed up for one year after the acute ischemic event. The one-year follow-up consisted of a telephone questionnaire whereas at 6 months, a more detailed examination was performed. At 6 months of follow-up, we performed a clinical examination, electrocardiography, an echocardiographic evaluation and blood harvesting to assess specific biomarkers (miRNAs).

During this one year of follow-up, we assessed the occurrence of MACE. In this study, MACE was defined as death from cardiovascular causes, heart failure requiring hospital admission or repeat PCI/CABG due to ischaemia/infarction (in concordance with previous trials [57]).

It is important to mention that all patients received optimal medical therapy at hospital discharge, according to current clinical practice guidelines [56].

### 2.7. Statistical Analysis

We performed the statistical analysis using SPSS software (IBM SPSS Statistics v.22.0, IBM Corp., Armonk, NY, USA) and GraphPad software (GraphPad Prism 9.0.0, San Diego, CA, USA). We presented the categorical data as percentages and the continuous variables as means. A Kolmogorov–Smirnov test and Mann–Whitney U-test were used for normal and non-normal distribution of data, respectively. Student t and X^2^ tests were used to compare continuous and categorical variables. To determine the predictors of MACE, we performed a Cox univariate regression, further incorporating the statistically significant variables in a multivariate analysis. An ROC analysis (receiver operating curve) was used to determine the AUC (area under the curve) representing the predictive power of the tested parameters. We also determined cut-off values for the significant variables using the Youden index. To further test the added values of miRNAs over myocardial work indices, we constructed prediction models and compared their statistical power using the C statistic and Akaike information criterion (AIC). We considered *p* under 0.05 as statistically significant.

## 3. Results

### 3.1. Characteristics of the Study Population

We included 50 young patients (<55 years), mean age of 44.78, with STEMI treated by primary PCI and 10 healthy control subjects (for miRNA results validation). Patients were divided into MACE group (9 cases, accounting for 18%) and non-MACE group (41 cases, accounting for 82%). The baseline characteristics of the entire study group divided according to the presence or absence of MACE are detailed in Table 1.

### 3.2. Echocardiographic Parameters

Echocardiographic parameters measured at baseline are reported in Table 2. Patients with MACE at follow-up had lower 2D LVEF (32.88 ± 5.79 vs. 43 ± 6.6 *p* = 0.000), more impaired LVGLS (−8.85 ± 1.58 vs. −13.8± 2.8, *p* < 0.0001) and higher 2D LVEDV (118.55 ± 29.43 vs. 99.26 ± 22.27, *p* = 0.031) and 2D LVESV (81.77 ± 25.36 vs. 54.87 ± 16.55, *p* = 0.013) at baseline.

Regarding medical treatment, there was no significant difference between MACE and no-MACE groups.

Echocardiography was performed both at baseline and at 6 months of follow-up. The echocardiographic parameters measured at follow-up are depicted in Table S1. Figure S1 (Supplementary Materials) represents the evolution of the myocardial work indices from baseline to follow-up. GWI, GCW and GWW appear to improve over time, but no significant change is observed in GWW.

### 3.3. miRNAs

Patients with STEMI had significantly higher levels of miRNA when compared to the control group (*p* < 0.005). All of the miRNAs associated with cardiovascular disease tested in this study were significantly upregulated in STEMI compared to the control group: miR-233-3p (*p* = 0.04), miR-142-3p (*p* = 0.009), miR-155-5p (*p* = 0.001), miR-486-5p (*p* = 0.001), miR-125a-5p (*p* = 0.013) and miR-146a-5p (*p* = 0.029). miRNA levels at baseline and at 6 months of follow-up are depicted in Figure S2.

We tested the correlations between miRNAs and cardiovascular risk factors (smoking, hypertension, dyslipidaemia and diabetes)—no significant correlations were observed. Interestingly, miR-125a-5p inversely correlated with age (Pearson −0.380, *p* = 0.006) and miR-142-3p with gender (Pearson −0.359, *p* = 0.01). miR-223-3p and miR-146a-5p both correlated with the Killip class (Pearson 0.291, *p* = 0.040 for miR-223-3p and Pearson 0.564, *p* = 0.000 for miR-146a-5p).

**Figure 2:**
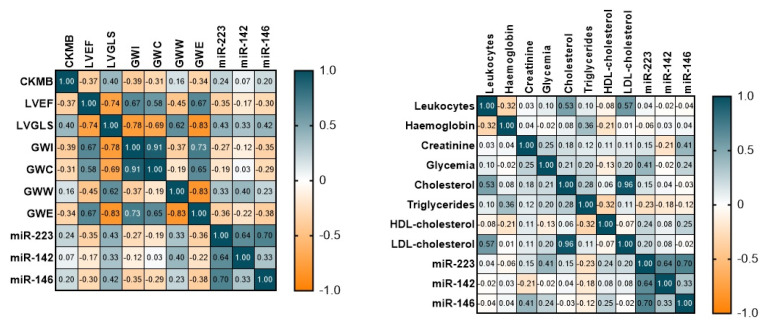
Correlation matrix: correlation between echocardiographic, biochemical parameters and miRNAs.

We also evaluated the correlation between echocardiographic parameters and miRNAs: the LV GLS values correlated with miR-223-3p (Pearson 0.429, *p* = 0.015); miR-142-3p (Pearson 0.335, *p* = 0.018); miR-4865p (Pearson 0.329, *p* = 0.02) and miR-146a-5p (Pearson 0.417, *p* = 0.003). The 2D LVEF values inversely correlated with miR-223-3p (Pearson −0.352, *p* = 0.012) and miR-146a-5p (Pearson −0.299. *p* = 0.035). miR-146a-5p inversely correlated with myocardial work indices—GWI (Pearson −0.347, *p* = 0.014), GCW (Pearson −0.288, *p* = 0.042) and GWE (Pearson = −0.378, *p* = 0.007). A few of the correlations are depicted below (Figure 2).

### 3.4. Clinical End Points—MACE

We divided the study cohort in two groups considering the presence or absence of MACE at the one-year follow-up (see Table 1 and Table 2 for detailed information). MACE occurred in 18% of the studied patients—12% readmissions for heart failure, 5% requiring PCI and 2% cardiovascular deaths.

No significant differences between the two groups regarding cardiovascular risk factors, clinical or angiographic characteristics were observed. There were a few differences in laboratory data—patients with MACE had higher CK-MB levels (*p* = 0.002) and glycaemic levels (*p* = 0.047). As expected, patients with MACE had a higher Killip class.

Table S2 depicts the associations between the echocardiographic parameters and the occurrence of MACE at follow-up (determined by a Cox regression analysis). We concluded that patients with MACE at follow-up had lower 2D and 3D LVEF, higher LV volumes, higher left ventricular filling pressures and more impaired LV strain.

#### 3.4.1. Myocardial Work Indices as Predictors of MACE

All four myocardial work indices (baseline values) were independent predictors for MACE at follow-up. They remained significant MACE predictors after adjustment for age, gender, 2D LVEF, LV dispersion and LV GLS. Moreover, after checking for collinearity, we obtained that GWI, GWE and GCW had a better predictive value as a group than separately. In the multivariate Cox regression analysis of the three variables, only GWI (*p* = 0.022, Wald 5.27) and GWE (*p* = 0.010, Wald 6.55) contributed significantly to the model—Table S3.

**Figure 3:**
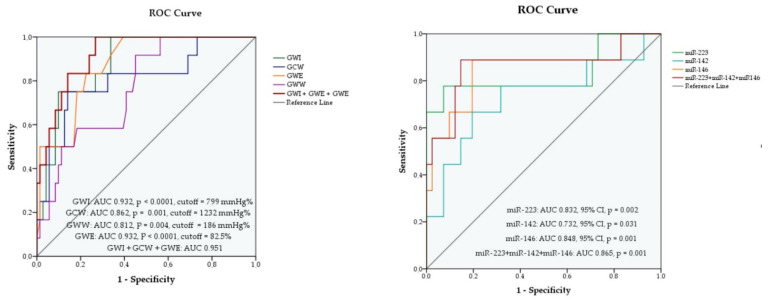
ROC curve analysis for baseline values of myocardial work indices (**left**) and miRNAs (**right**) as predictors of MACE at follow up.

The four myocardial work indices had good prediction potential for MACE, with an AUC greater than 0.7 in the ROC curve analysis as follows: AUC 0.932 (95% CI), *p* < 0.0001 for GWI; AUC 0.862 (95% CI), *p* = 0.001 for GCW; AUC 0.812 (95% CI), *p* = 0.004 for GWW and AUC 0.932 (95% CI), *p* < 0.0001 for GWE, as shown in Figure 3. Out of the three variables, GWI and GWE proved to have the best predictive value for MACE, both with AUC 0.932, *p* < 0.0001. The highest AUC was obtained for the combination of the three parameters GWI, GCW and GWE, with AUC 0.951, *p* < 0.0001, proving a better prediction ability than each variable separately (Figure 3 and Figure S3).

For each variable we determined a cut-off value, based on the maximum value of the Youden index as follows: 799 mmHg% (sensitivity 88.9%, specificity 92.7%) for GWI, 1232 mmHg% (sensitivity 88.9%, specificity 82.9%) for GCW, 186 mmHg% (sensitivity 77.8%, specificity 63.4%) for GWW and 82.5% (sensitivity 88.9%, specificity 70.7%) for GWE as shown in Table S4.

For comparison, we performed an ROC analysis for classical echocardiographic MACE predictors: LV GLS (AUC 0.924, *p* < 0.0001), LV mechanical dispersion (AUC 0.764, *p* = 0.014) and 3D LVEF (AUC 0.888, *p* < 0.0001). Considering this, GWI and GWE are better at predicting an outcome in this patient group than the standard parameters.

#### 3.4.2. Association of miRNA Levels with Cardiovascular Outcome

**Figure 4:**
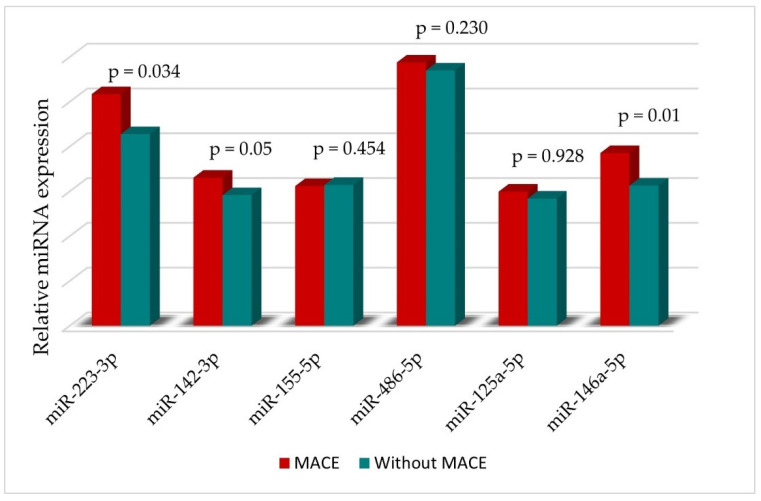
miRNA expression (log 10) at baseline in the entire population and divided according to the occurrence of MACE.

We observed that the expression levels of the three miRNAs were higher in patients in the MACE group compared with the non-MACE group. STEMI patients with MACE had a higher expression of miR-223-3p and miR-146a-5p plasma levels at baseline than those without MACE (Figure 4).

Using a Cox binary univariate regression analysis, we tested the predictive potential of these markers and found that only three of the circulating miRNAs were significantly associated with the primary endpoint (MACE) in young STEMI patients: miR-223-3p (*p* = 0.000), miR-142-3p (*p* = 0.022) and miR-146a-5p (*p* = 0.000). Introducing the variables in a multivariable Cox regression model (chi-square = 13.792, *p* of model = 0.003), only miR-146a-5p remained independently associated with MACE (*p* = 0.012, Wald 6.37).

We then estimated the prediction potential of the three plasma miRNAs for MACE in patients with STEMI, using a receiver operator curve analysis (ROC). According to this analysis, the three miRNAs had good prediction abilities for MACE with an AUC greater than 0.7: AUC 0.832 (95% CI), *p* = 0.002 for miR-223-3p; AUC 0.732 (95% CI), *p* = 0.031 for miR-142-3p and AUC 0.848 (95% CI), *p* = 0.001 for miR-146a-5p (Figure 3). For each variable we determined a cut-off value, based on the maximum value of the Youden index as follows: 146,133 (sensitivity 77.8%, specificity 87.8%) for miR-233-3p, 1115 (sensitivity 77.8%, specificity 68.3%) for miR-142-3p and 4155 (sensitivity 88.9%, specificity 80.5%) for miR-146a-5p, as shown in Table S5. Out of the three miRNAs, miR-146a-5p proved to have the best predictive value for MACE. We also assessed their predictive value as a group and obtained an AUC of 0.865, 95% CI, *p* < 0.0001 Table S5. It is worth mentioning that in the Cox multivariate logistic regression analysis with the combination of the three miRNAs, miR-146a-5p had a significant contribution to the model with *p* = 0.012.

**Figure 5:**
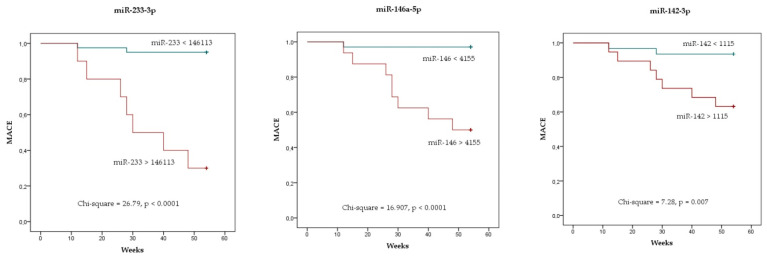
Kaplan–Meyer analysis curves showing the risk of MACE stratified by miR-223 (**left**), miR-146 (**middle**) and miR-142 (**right**). Cut-off value for each variable was calculated by ROC analysis.

A Kaplan–Meier analysis showed the survival curves for the risk of MACE with respect to miR-223-3p, miR-142-3p and miR-146a-5p expression. Patients with higher miRNA levels at baseline had a higher probability of MACE at follow-up (details below in Figure 5).

#### 3.4.3. Comparison between the Prognostic Power of Myocardial Work Indices and miRNAs

Myocardial work indices (GWI, GWC and GWE) proved to have a better prognostic potential than the three miRNAs (AUC = 0.951 for myocardial work indices and AUC = 0.862 for miRNAs) (Figure S3). However, their combination had better prognostic power than each separately as shown in the next paragraph.

#### 3.4.4. Incremental Prognostic Value of Circulating miRNAs over Myocardial Work Indices

To further test the added value of miRNAs as prognostic markers, we built two logistic models: model 1 included myocardial work indices (GWI, GCW, GWE) and model 2 included sex, age and myocardial work indices. We determined the ability of each miRNA and of the combination of miRNAs to improve the predictive value of the three models.

Each of the three miRNAs and their associations added value to all the proposed predictive models as depicted below (Table 3 and Table S6).

To support our findings, we compared the prediction potential of the combination of the three miRNAs with myocardial indices and the combination of the three miRNAs with LVGLS and 2D LVEF and in each case, the combination of miRNAs with myocardial work parameters yielded the best AIC and C-statistic.

## 4. Discussion

This was the first study to assess the potential prognostic value of a group of miRNAs together with new echocardiographic parameters (myocardial work indices) in predicting adverse events/MACE in a group of young patients with STEMI.

We focused our attention on young adults with STEMI considering its increasing prevalence in this group. The diagnosis of AMI at a younger age exerts a significant health, socioeconomic and psychological burden not only upon the patient but also upon the entire community. Finding a good prognostic marker might help improve the stratification risk and prognosis in these patients.

### 4.1. Myocardial Work Indices as MACE Predictors in STEMI

Recently, the LV myocardial work analysis was proposed as a new method for evaluation of LV systolic function. Combining LV pressure–strain loops derived from GLS and blood pressure measurements, this new method takes into account the loading conditions [14]. Even though 2D LVEF and GLS are currently the most used echocardiographic parameters for LV systolic function assessment, myocardial work parameters have proved to be less load-dependent, therefore more reliable [14].

Previous studies evaluated the clinical applications of this new method of assessing systolic function in various cardiovascular diseases. In ischaemic heart disease, myocardial work indices have proved to have good diagnostic value in a few studies. Guo et al. demonstrated that regional myocardial work measured by echocardiography exhibited a good diagnostic value in detecting significant myocardial ischaemia compared to the standard fractional flow reserve approach (measured invasively in the catheterisation laboratory) [58]. The use of regional GWE was able to identify at baseline CAD patients with critical coronary artery stenosis before invasive angiography with excellent performance (AUC = 0.92) in a trial from 2021 [59].

In our study cohort of young STEMI patients treated by primary PCI, we found that myocardial work parameters measured noninvasively were independent predictors of MACE at one-year follow-up.

We first assessed the evolution of MW in time in the entire cohort. At follow-up, we observed that GWI, GCW and GWE values were higher than baseline (1180.29 at baseline vs. 1138.4 at follow-up for GWI, *p* = 0.007; 1493 at baseline vs. 1663.29 at follow-up for GCW *p* = 0.089, 87 at baseline vs. 90 at follow-up, for GWE *p* = 0.004) but with little to no changes in GWW (182.88 at baseline vs. 181.51 at follow-up, *p* = 0.943) results, consistent with a recent study [60]. We then compared the evolution in time of myocardial work parameters between the MACE and non-MACE groups. While in the non-MACE group the myocardial work indices improved significantly at follow-up, in the MACE group they did not. This emphasises the potential prognostic potential of GWI, GCW and GWE.

Butcher et al. found GWI as independently associated with all-cause mortality at 6 months of follow-up after STEMI, providing an incremental prognostic value over LVEF and a minor incremental prognostic value over LV GLS in a study of 179 patients with reduced left ventricular ejection fraction [21]. While Lustosa et al. demonstrated the prognostic power of GWE at 80 months after STEMI in 507 patients [20], in another study GCW proved to be an independent predictor of segmental and global LV remodelling in patients with anterior MI treated by primary PCI [61]. Taking these trials into consideration, an evaluation of all myocardial work indices as MACE predictors in young patients with STEMI is currently lacking.

In our study on young STEMI patients, GWI, GWE and GCW had significantly lower values in the MACE group compared to the group without MACE. In an ROC analysis, the GWI, GWE but also the GCW baseline values proved to have good prediction abilities for MACE at one-year follow-up after STEMI with: AUC 0.932, *p* < 0.0001 for GWI; AUC 0.862, *p* = 0.001 for GCW; AUC 0.812, *p* = 0.004 for GWW and AUC 0.932, *p* < 0.0001 for GWE. GWI and GWE proved to be the best predictors for MACE among the myocardial indices. Moreover, we demonstrated that GWI, GWE and GCW had a better predictive value as a group than separately with AUC = 0.951, *p* < 0.0001 (Figure 3). It is worth mentioning that GWI and GWE proved to be better predictors compared with other standard echocardiographic parameters (LVEF, LVGLS and mechanical dispersion).

STEMI patients with lower values of GWI, GCW and GWE at baseline proved to have a higher probability of developing adverse events in time.

### 4.2. MIRNAs as MACE Predictors in STEMI

MiRNAs have been proved to participate in many cardiovascular disorders and pathological processes of cardiovascular diseases, such as atherosclerosis, coronary artery disease, heart failure, cardiac remodelling, arrhythmias and myocardial ischaemia [62,63].

Recent research emphasises the rising potential of miRNAs as novel biomarkers in ischemic heart disease, and its extreme manifestation, acute coronary syndrome [64].

In our study, higher circulating levels of miR-233-3p, miR-142-3p and miR-146a-5p measured within 48 h from symptom onset in a group of young patients were identified as independent predictors of future adverse cardiac events one year after STEMI.

miR-223-3p levels at baseline were higher in STEMI patients compared to controls. This could be explained by the fact that this is a cardiac-specific miRNA, moderately expressed in cardiomyocytes [65]. Previous studies have found that miR-223-3p is strongly upregulated during the early stages of myocardial infarction before the elevation of troponin I and CK-MB [66] with a higher expression in the ischemic compared to the normal myocardium. Our findings are in agreement with previous trials that demonstrated its connection to multiple pathological processes including atherosclerosis (miR-233-3p levels are significantly elevated in patients with atherosclerosis) [67]; it also has a role in platelet activation [68], the modulation of cholesterol homeostasis [69] and the transport function of lipoproteins [70]. Previous trials have stated its role as a marker of atherosclerotic plaque instability in patients with CAD [33] and a predictor of thrombotic events that could be used for ischemic risk stratification after AMI [34]. All these data support its potential role in STEMI patients.

We obtained that a value of miR-233-3p expression over the cut-off value (AUC 0.832, 95% CI, *p* = 0.002) at baseline was an independent predictor of MACE. Furthermore, in the Kaplan–Meier analysis, patients with higher levels of miR-223-3p in the acute setting had a higher probability of developing MACE at follow-up (Log rank chi-square 26.46, *p* < 0.0001). These results are consistent with Schulte et al. [71], who reported that increased circulating miR-223-3p in coronary artery disease could be used to predict cardiovascular death risk for patients, in particular for patients with ACS, in a study on 873 patients. Moreover, elevated levels of miR-223-3p positively associated with the severity of coronary atherosclerotic lesions evaluated by Gensini scores in a study from 2018, supporting its role as prognostic marker [72].

miR-146a-5p has proved to be upregulated in atherosclerotic plaques [73] and also in patients with coronary artery disease [38,39]. Takahashi et al. discovered that circulating levels of miR-146a and miR-146b (related to inflammation) were elevated in patients with CAD (compared with patients without CAD) [74]. Supporting these data, higher miR-146a-5p levels were encountered in patients with ACS compared with unstable angina [75,76]. Moreover, Xiao et al. found that miR-146a might serve as a marker for MACE in STEMI patients [77].

We also tested its predictive power in STEMI patients and observed a correlation between high levels of miR-146a-5p and MACE. In Cox univariate regression analysis, it proved to be a predictor of MACE at one year follow up. ROC curve analysis confirmed that-AUC 0.848 (95% CI), *p* = 0.001. miR-146a-5p had the highest AUC between the three miRNAs, therefore the greatest value as potential predictor of adverse events.

miR-142-3p, known to be involved in atherosclerosis [55] and ischemic heart disease [35] proved to be an independent prognostic marker of adverse outcome in our group of young patients with STEMI. Its prognostic value was tested using an ROC curve analysis, where we obtained an AUC of 0.732 (95% CI), *p* = 0.031, and determined an optimal cut-off value of 1115 (sensitivity 77.8%, specificity 68.3%) for MACE prediction. In good agreement with these data, a strong predictive potential for subsequent cardiovascular events was proved in our previous study on multiple vascular atherosclerotic patients with peripheral artery disease [55]. It is worth mentioning that among the three tested miRNAs, miR-142-3p had the lowest predictive power for MACE in a Cox univariate regression and in an ROC curve analysis (AUC).

STEMI patients with miRNAs values higher than the cut-off points (previously obtained by ROC analysis) appeared to have a higher probability of developing MACE in a Kaplan–Meier analysis. The increased plasma levels of the three miRNAs could be used to predict unfavourable outcomes in STEMI patients.

### 4.3. miRNAs and Myocardial Work Parameters

As far as we know, this is the first study to assess the power of a group of miRNA and myocardial work indices to predict the occurrence of MACE after STEMI in young patients. In this study, we found that both miRNAs and myocardial work parameters had good prognostic power in the studied population.

miR-223-3p, miR-142-3p and miR-146a-5p had incremental prognostic value over myocardial work indices and together, they could better predict unfavourable outcomes than each separately. The addition of each of the three miRNAs over myocardial work indices (GWI, GCW and GWE) in a Cox multivariate regression analysis, yielded a higher AIC and C-statistic than those of the myocardial indices alone (the best model between two was chosen based on the likelihood ratio test).

Our findings hold great potential for future treatment monitoring and personalised patient management according to risk stratification.

### 4.4. Limitations

Our study had several limitations. The first limitation is the small sample population (due to the age threshold of the study group and COVID 19 pandemic)—the performance and precision of predictions may have been affected. Second, another limitation is related to the lack of a comparison group of older STEMI patients. Third, at one year, we only had a telephonic follow-up available due to COVID-19 pandemic restrictions.

Larger further studies are required to validate our findings.

Despite these limitations, our study holds great potential, considering the obtained results and their further possible utility as MACE predictors in a young population.

## 5. Conclusions

Myocardial work indices and the three miRNAs tested in this study (miR-223-3p, miR-142-3p and miR-146a-5p) have a promising prognostic potential, as independent markers and also as a group, in young STEMI patients. The complementary use of miRNAs has incremental prognostic value over the tested echocardiographic parameters (GWI, GCW and GWE).

miRNAs together with myocardial work indices are better at predicting MACE than each separately and have the potential to be used as prognostic biomarkers; this might further facilitate risk stratification and the guidance of clinical care, improve secondary prevention and even lower cardiovascular mortality in young patients after STEMI.

## Figures and Tables

**Table 1:** Baseline characteristics of the entire study population divided in two subgroups according to the occurrence of MACE during follow up.

	Study Population (*n* = 50)	MACE (*n* = 9)	Without MACE (*n* = 41)	*p* Value
Clinical characteristics				
Age (years)	44.7 ± 5.62	44 ± 3.78	45 ± 5.98	0.99
Systolic blood pressure (mmHg)	119.54 ± 16.66	120.44 ± 20.35	119.34 ± 16.03	0.859
Cardiovascular risk factors				
Smoking	86%	77.8%	87%	0.370
Obesity	22%	0%	24.2%	0.109
Hypertension	46%	33.3%	48.8%	0.321
Dyslipidaemia	75.6%	77.8%	82.9%	0.517
Diabetes	17.1%	11.1%	12.2%	0.707
Metabolic syndrome	12.2%	40%	17.6%	0.248
Clinical presentation				
Killip class ≥2	17%	77.7%	4.8%	**<0.0001**
Angiographic characteristics				
LAD	48%	77.8%	41.5%	0.069
RCA	48%	77.8%	41.5%	0.67
LCX	24%	0%	29.3%	0.092
Multivessel CAD	34.6%	22.2%	77.8%	0.459
Occluded artery	53.8%	66.7%	33.3%	0.713
Symptom to balloon time	6.6 ± 5.31	7.5 ± 5.44	6.55 ± 7.26	0.692
Laboratory characteristics				
WBC count, × 10^3^/mm^3^	11,260 ± 3628	16,088.89 ± 3417.39	13,807 ± 1711.6	0.695
Haemoglobin, g/dL	14.06 ± 1.44	13.41 ± 1.24	14.02 ± 2.81	0.411
Creatinine (mg/dL)	0.83 ± 0.23	0.90 ± 0.40	0.82 ± 0.17	0.38
Glycaemia (mg/dL)	118.02 ± 38.62	136.22 ± 48.41	108.69 ± 33.36	**0.047**
Cholesterol (mg/dL)	217.21 ± 64.36	199.40 ± 67.15	224.08 ± 52.61	0.347
Triglycerides (mg/dL)	202.37 ± 181.288	125.47 ± 72.66	151.61 ± 71.65	0.321
HDL-cholesterol	28.08 ± 11.95	26.47 ± 12.30	28.47 ± 12.01	0.482
LDL-cholesterol	159.30 ± 53.95	147.84 ± 63.52	162.09 ± 51.69	0.658
Peak CK-MB (U/L)	251.58 ± 211.26	479.67 ± 296.824	198.00 ± 144.125	**0.022**

**Table 2:** Echocardiographic parameters at baseline in the entire study population and divided in two subgroups according to the occurrence of MACE.

	Population	MACE (*n* = 9)	Without MACE (*n* = 41)	*p* Value
2D LVEDV (mL)	102.74 ± 24.54	118.55 ± 29.43	99.26 ± 22.27	**0.031**
2D LVEDV (mL/mp)	53.97 ± 12.6	64.18 ± 13.91	51.75 ± 11.28	**0.06**
2D LVESV (mL)	59.72 ± 20.91	81.77 ± 25.36	54.87 ± 16.55	**0.013**
2D LVESV (mL/mp)	59.72 ± 20.91	81.77 ± 25.36	54.87 ± 16.55	**0.013**
2D EF (%)	41.94 ± 7.07	32.88 ± 5.79	43 ± 6.6	**<0.0001**
3D LVEDV (mL)	113.46 ± 24.46	127.66 ± 28.48	110.34 ± 22.7	**0.053**
3D LVEDV (ml/mp)	59.77 ± 13.02	69.24 ± 13.45	57.69 ± 12.36	**0.016**
3D LVESV (mL)	65.74 ± 21.15	87 ± 25.91	61.07 ± 17.02	**0.001**
3D LVESV (mL/mp)	34.67 ± 11.34	47.13 ± 12.73	31.93 ± 9.08	**<0.0001**
3D LVEF (%)	40.02 ± 8.05	33 ± 6.55	45.24 ± 6.5	**<0.0001**
LV GLS	−12.93 ± 2.2	−8.85 ± 1.58	−13.8 ± 2.8	**<0.0001**
LV mechanical dispersion	72.57 ± 26.49	93.11 ± 29.36	68.06 ± 23.9	**0.009**
E/e’ (LV filling pressure)	8.2 ± 2.92	10.68 ± 2.01	7.59 ± 2.03	**<0.0001**
Myocardial work indices				
LV GWI, mmHg%	1089.66 ± 318.97	1167.07 ± 295.67	737 ± 124.24	**<0.0001**
LV GCW, mmHg%	1430.54 ± 325.37	1499.68 ± 304.01	1115.55 ± 224.06	**0.001**
LV GWW, mmHg%	193.14 ± 105.84	172.75 ± 96.3	286 ± 102.07	**0.003**
LV GWE, %	86.12 ± 6.55	87.95 ± 5.53	77.77 ± 3.8	**<0.0001**

**Table 3:** C-statistics, AIC and likelihood ratio test for incremental predictive values of MACE obtained for model 1 by addition of miRNAs.

	*p* Value	Statistic log Likelihood Ratio	AIC	C-Statistic	Likelihood Ratio Test
Model 1 (GWI + GCW + GWE)	*p* < 0.0001	27.577	44.07	0.938 (0.884–0.991)	
+miR 223-3p	*p* < 0.0001	33.064	40.53	0.9504 (0.909–0.991)	0.0186
+miR 142-3p	*p* = 0.0024	35.027	38.11	0.9504 (0.905–0.995)	0.0048
+miR 146a-5p	*p* < 0.0001	34.674	38.58	0.9603 (0.932–0.988)	0.0062
+miR 223-3p + miR 142-3p	*p* < 0.0001	37.049	38	0.9553 (0.9165–0.9942)	0.0067
+miR 142-3p + miR 146a-5p	*p* < 0.0001	42.719	31.19	0.975 (0.949–1.001))	0.0002
+miR 223-3p + miR 146a-5p	*p* < 0.0001	34.934	40	0.960 (0.9329–0.9877)	0.0216
+miR 223 + miR-142 + miR-146	*p* < 0.0001	44.068	31	0.9777 (0.952–1.003)	0.0003

## Data Availability

The data will be available based on reasonable request.

## Supplementary Materials

The following are available online at https://www.mdpi.com/article/10.3390/diagnostics12081946/s1, Table S1: Echocardiographic parameters at baseline and at follow-up in the entire population, Figure S1: Changes in myocardial work indices after STEMI (baseline and 6-month follow-up): GWI (upper left), GCW (upper right), GWW (lower left), GWE (lower right), Figure S2: miRNA levels at baseline in the study population and in the control group, Table S2: Cox univariate regression for the occurrence of MACE, Table S3: Univariate and multivariate regression analysis of myocardial work indices (at baseline) as predictors of MACE, Table S4: ROC analysis for myocardial work indices at baseline as predictors for MACE at follow-up, Table S5: ROC analysis performance of MACE prediction using miRNAs baseline values, Figure S3: ROC analysis for Cox regression models of myocardial work indices and miRNAs (baseline values) as predictors for MACE at follow-up in young STEMI patients. Table S6: C-statistics, AIC and likelihood ratio test for incremental predictive values of MACE obtained for model 2 by adding miRNAs.

## Abbreviations

ACS	Acute Coronary Syndrome
AIC	Akaike information criterion
AMI	acute myocardial infarction
AUC	area under the curve
CAD	coronary artery disease
CI	confidence intervals
CK_MB	creatine kinase-MB
EDTA	ethylenediaminetetraacetic acid
GCW	global contraction work
GLS	global longitudinal strain
GWE	global work efficiency
GWI	global work index
GWW	global wasted work
HDL	high-density lipoprotein
LAD	left anterior descending artery
LCX	left circumflex artery
LDL	low-density lipoprotein
LV	left ventricular
LVEDV	LV end diastolic volume
LVEF	left ventricular ejection fraction
LVESV	LV end-systolic volume
MACE	major adverse cardiac events
MW	myocardial work
OR	odds ratio
PCI	percutaneous coronary intervention
RCA	right coronary artery
ROC	receiver operating characteristic
RT-PCR	reverse transcription polymerase chain reaction
STEMI	acute ST elevation myocardial infarction
WBC	white blood cells
