# Supplementary material for: miR-146a-5p, miR-223-3p and miR-142-3p as Potential Predictors of Major Adverse Cardiac Events in Young Patients with Acute ST Elevation Myocardial Infarction—Added Value over Left Ventricular Myocardial Work Indices
Source: Diagnostics (Basel). 2022 Aug 12;12(8):1946. doi: 10.3390/diagnostics12081946 (PMC9406722; doi:10.3390/diagnostics12081946)
Supplement: Supplementary file 1 [file diagnostics-12-01946-s001.zip › diagnostics-1836350-supplementary.pdf]

diagnostics

MDPI

SUPPLEMENTARY MATERIAL FOR MANUSCRIPT

# miR-146a-5p, miR-223-3p and miR-142-3p as Potential Predictors of Major Adverse Cardiac Events in Young Patients with Acute ST Elevation Myocardial Infarction - Added Value over Left Ventricular Myocardial Work Indices

Sorted by the order of appearance in main manuscript:

**Table S1.** Echocardiographic parameters at baseline and at follow up in the entire population.

| | Population at baseline | Population at follow up | P value |
|---|---|---|---|
| 2D LVEDV (ml) | 103.51±23.67 | 114.33±31.08 | **0.002** |
| 2D LVESV (ml) | 61.27±22.26 | 60.63±27.04 | **0.772** |
| 2D EF (%) | 42.08±7.721 | 47.59±8.5 | **0.000** |
| 3D LVEDV (ml) | 110.85±24.4 | 121.5±26.85 | 0.000 |
| 3D LVESV (ml) | 64.82±22.67 | 63.17±23.55 | 0.256 |
| 3D LVEF (%) | 42.78±8.23 | 49.17±7.71 | **0.000** |
| LV GLS | -13.5±3.47 | -14.9±3.65 | **0.011** |
| LV mechanical dispersion | 67.84±19.27 | 65.67±30.91 | **0.656** |
| E/e’ (LV filling pressures) | 8.57±2.72 | 8±3.14 | **0.248** |
| GWI | 1180.29±326.58 | 1318.4±360.2 | **0.007** |
| GCW | 1493±335.65 | 1663.29±412.47 | **0.015** |
| GWW | 182.88±109.62 | 181.51±95.44 | **0.943** |
| GWE | 87.55±6.95 | 90±6.49 | **0.004** |

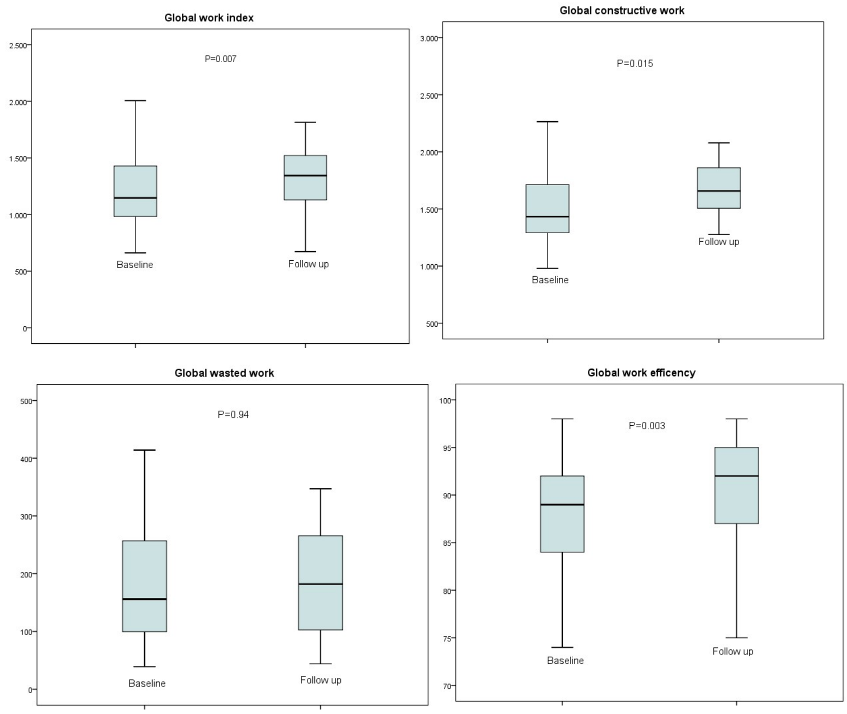


**Figure S1.** Changes in myocardial work indices after STEMI (baseline and 6 months follow up): GWI (upper left), GCW (upper right), GWW (lower left), GWE (lower right).

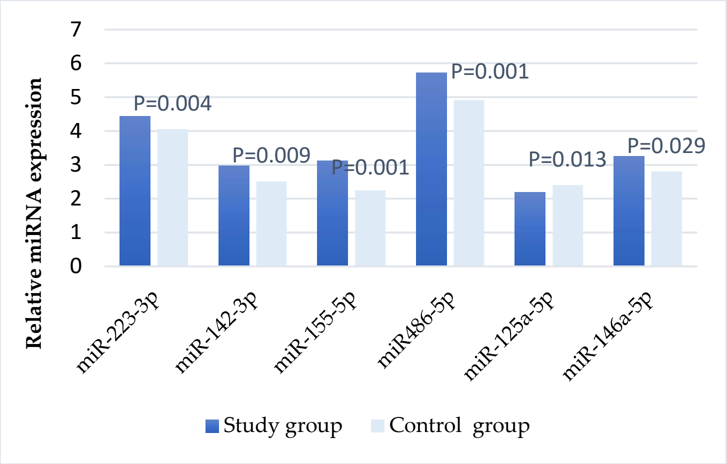


**Figure S2.** miRNA levels at baseline in the study population and in the control group.

**Table S2.** COX univariate regression for the occurrence of MACE.

| | Parameters at baseline | OR | CI | P value |
|---|---|---|---|---|
| *Clinical characteristics* | Age | 1.002 | 0.967-1.002 | 0.967 |
| | Sex | 3.65 | 0.912-14.621 | 0.067 |
| | Killip class | 3.487 | 1.984-6/127 | **<0.0001** |
| *Biological parameters* | Leukocytes | 1 | 1.000-1.000 | 0.698 |
| | Haemoglobin | 0.929 | 0.779-1.108 | 0.464 |
| | Glycemia | 0.072 | 0.999-1.022 | 0.111 |
| | Creatinine | 3.032 | 0.173-53.026 | 0.447 |
| | Cholesterol | 0.992 | 0.98-1.005 | 0.227 |
| | CK-MB max | 1.005 | 1.003-1.008 | **<0.0001** |
| *Echocardiographic parameters* | 2D LVEF | 0.816 | 0.730-0.912 | **<0.0001** |
| | 2D LVEDV indexed | 1.085 | 1.027-1.146 | **0.004** |
| | 2D LVESV indexed | 1.124 | 1.060-1.192 | **<0.0001** |
| | GLS LV | 2.34 | 1.533-3.587 | **<0.0001** |
| | 3D LVEF | 0.775 | 0.678-0.886 | **<0.0001** |
| | 3D LVEDV indexed | 1.076 | 1.017-1.137 | **0.01** |
| | 3D LVESV indexed | 1.127 | 1.058-1.200 | **<0.0001** |
| | E/e′ ratio | 1.855 | 1.379-2.494 | **<0.0001** |
| | GWI | 0.992 | 0.988-0.996 | **<0.0001** |
| | GCW | 0.995 | 0.991-0.998 | **<0.0001** |
| | GWW | 1.0008 | 1.002-1.014 | **0.007** |
| | GWE | 0.810 | 0.716-0.917 | **<0.0001** |

**Table S3.** Univariate and multivariate regression analysis of myocardial work indices (at baseline) as predictors of MACE.

| | **Univariate regression analysis** | | | | **COX multivariate regression analysis** | | |
|---|---|---|---|---|---|---|---|
| | **Chi square** | **Wald** | **OR** | **P value** | **Wald** | **P value** | **Chi-square for model** |
| **GWI** | 21.065 | 13.9 | 0.992 | 0.000 | 5.909 | **0.015** | |
| **GCW** | 14.143 | 9.131 | 0.995 | 0.003 | 0.452 | 0.501 | 27.57 |
| **GWW** | 7.435 | 7.243 | 1.008 | 0.007 | - | - | P value **0.0001**<br>**C statistic 0.938, CI 0.884-0.991** |
| **GWE** | 19.45 | 15.408 | 0.777 | 0.000 | 4.118 | **0.042** | |

**Table S4.** ROC analysis for myocardial work indices at baseline as predictors for MACE at follow up.

| | *AUC* | *P value* | *Cut off value* | *Sensibility* | *Specificity* |
|---|---|---|---|---|---|
| **CWI** | 0.932 | 0.000 | 799 | 88.9 | 92.7 |
| **GCW** | 0.862 | 0.001 | 1232 | 88.9 | 82.9 |
| **GWW** | 0.812 | 0.004 | 186 | 77.8 | 63.4 |
| **GWE** | 0.932 | 0.000 | 82.5 | 88.9 | 80.5 |

**Table S5.** ROC analysis -performance of MACE prediction using miRNAs baseline values.

| | **AUC** | **P value** | **Cut off value** | **Cut off value (Log 10)** | **Sensibility** | **Specificity** |
|---|---|---|---|---|---|---|
| **miR-223-3p** | 0.832 | 0.002 | 146113 | 5.164 | 77.8% | 87.8% |
| **miR-142-3p** | 0.732 | 0.031 | 1115 | 3.047 | 77.8% | 68.3% |
| **miR-146a-5p** | 0.848 | 0.001 | 4155 | 3.614 | 88.9% | 80.5% |
| **miR-223-3p + miR-142-3p + miR146a-5p** | 0.865 | 0.000 | | | | |

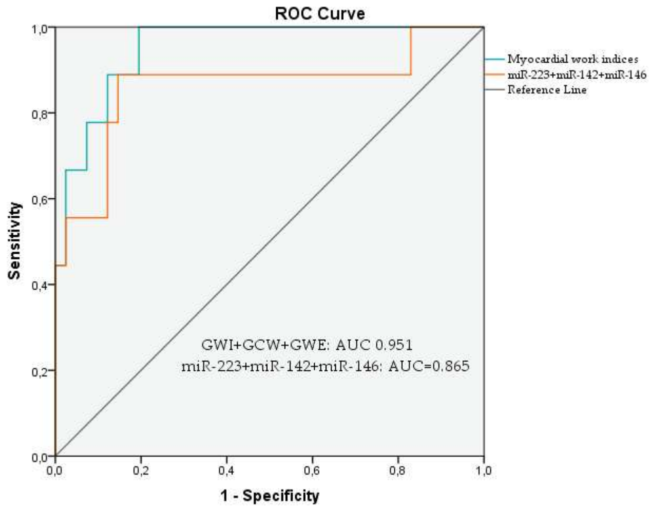


**Figure S3.** ROC analysis for Cox regression models of myocardial work indices and miRNAs (baseline values) as predictors for MACE at follow up in young STEMI patients.

**Table S6.** C-statistics, AIC and likelihood ratio test for incremental predictive values of MACE obtained for Model 2 by adding miRNAs.

| | **P value** | **Statistic log likelihood ratio** | **C-statistic** | **AIC** | **Likelihood ratio test** |
|---|---|---|---|---|---|
| Model 2 (age, sex, MW indices) | P<0.0001 | 30.377 | 0.945 (0.903-0.987) | 45.06 | |
| + miR-233-3p | P <0.001 | 35.928 | 0.9603 (0.919-1.001) | 41.47 | 0.0181 |
| + miR-142-3p | P<0.001 | 35.143 | 0.9504 (0.905-0.995) | 41.93 | 0.0235 |
| + miR-146a-5p | P<0.0001 | 42.512 | 0.977 (0.956-0.999) | 34 | 0.0003 |

The likelihood ratio test compares the fit of a model with miRNAs to model 1.
